# Cross-Kingdom Infection of Macrophages Reveals Pathogen- and Immune-Specific Global Reprogramming and Adaptation

**DOI:** 10.1128/mbio.01687-22

**Published:** 2022-07-12

**Authors:** Arjun Sukumaran, Brianna Ball, Jonathan R. Krieger, Jennifer Geddes-McAlister

**Affiliations:** a Department of Molecular and Cellular Biology, University of Guelph, Guelph, Ontario, Canada; b Bruker Canada, Inc. Milton, Ontario, Canada; c Canadian Proteomics and Artificial Intelligence Research and Training Consortium, Guelph, Ontario, Canada; Duke University Medical Center

**Keywords:** coinfection, quantitative proteomics, macrophages, pathogenesis, *Cryptococcus neoformans*, *Klebsiella pneumoniae*, cross-kingdom

## Abstract

The interactions between a host and microbe drive the health and disease status of the host. Of importance is the cause of dysbiosis in the presence of a pathogen, and critically, the relationship between the host and pathogen may evolve over time through response and adaptation. For immunocompromised individuals, dual infections are prevalent and contribute to disease severity and treatment options. Here, we explore the global reprogramming of host cells in response to immediate and established microbial infections with the human fungal pathogen Cryptococcus neoformans and the nosocomial bacterial pathogen Klebsiella pneumoniae. Using quantitative proteomics, we uncovered cross-kingdom protein-level changes associated with initial fungal infection, followed by a remarkable adaptation of the host and pathogen to a dormant state. This stabilization is disrupted over time upon bacterial infection, with the production of virulence-associated bacterial proteins and severely altered host response. We support our findings with the profiling of two major virulence determinants in C. neoformans, catalase and melanin, which demonstrate an interconnected regulation in response to both host defense and bacterial invasion. Overall, we report novel fungal and bacterial modulation of the host, including adaptation and stabilization, suggesting an opportunity to effectively treat dual infections by selectively targeting proteins critical to the host’s infection stage.

## INTRODUCTION

The study of the human microbiota and its role in infectious diseases is a rapidly expanding field. This interest is due to implications of the complex relationship between the host and its commensal community and how disruptions to this partnership may influence host responses to microbes. Recent exploration within the microbiome to focus on the impact of the fungus-derived mycobiome is further advancing the area of study and its role in modulating human health (1, 2). The composition of the lung mycobiome may influence the host immune response to be protected or vulnerable in the development of many types of diseases. For instance, the pulmonary mycobiome has been reported to play a role in the clinical outcome of established respiratory diseases, such as asthma, cystic fibrosis, and chronic obstructive pulmonary disease (3–6). Furthermore, fungal pulmonary infections are an emerging public health threat featuring staggeringly high mortality rates, poor diagnosis, and limited access to antifungal drugs (7). A predominant and widespread fungal pathogen is the environmental fungus Cryptococcus neoformans, responsible for causing life-threatening cryptococcal meningitis, which impacts 220,000 immunocompromised individuals annually (8). This opportunistic pathogen initiates infection upon inhalation of desiccated yeast cells from various environmental sources, leading to the establishment of a pulmonary infection. C. neoformans pathogenesis is equipped with crucial virulence factors, including a polysaccharide capsule, melanin, and extracellular enzymes that mitigate its ability to cause disease (9).

Bacterium-fungus interactions can dramatically shift the host response from infection to disease. This shift may relate to the production of virulence factors and pathogenic survival mechanisms during dysbiosis upon coinfections (e.g., microbiome disruption that may elicit pathogenic responses) (10). Commonly studied bimicrobial interactions include the human fungal pathogen Candida albicans with the bacterial pathogen Pseudomonas aeruginosa, a colonizer of the respiratory tract, within the context of coinfection during cystic fibrosis (11, 12). However, relatively few studies have focused on Cryptococcus*-*bacterium interactions, despite the unique ability of *Cryptococcus* to remain present within the lung for undetermined periods by transitioning into a dormant state (13). Notably, C. neoformans sensitivity to the murine lung microbiota induces a morphological yeast-to-Titan cell transition that can exacerbate host immune evasion and increase fungal dissemination (14). Thus, C. neoformans has a high likelihood of both transient and prolonged interactions with components of the human microbiota during both healthy and diseased states (15). For example, antagonistic interactions have been observed to occur between C. neoformans and P. aeruginosa via bacterium-fungal cell contact triggering the production of inhibitory anticryptococcal factors (16). Similarly, antifungal activity has also been reported from the bacterial pathogen Staphylococcus aureus during coculture with C. neoformans (17). Furthermore, fungal virulence factor production may be activated during coincubation with bacteria, such as in the presence of the opportunistic bacterial pathogen Klebsiella aerogenes, to produce dopamine, which serves as a substrate for cryptococcal melanin biosynthesis (18).

Another important consideration is the requirement for hospitalization of Cryptococcus-infected individuals for treatment (e.g., administration of antifungals), which increases the likelihood of exposure to hospital-acquired bacterial infections (19, 20). Such hospital-acquired or nosocomial infections are a serious public health concern due to considerably higher mortality rates and increased hospitalization demands. A common nosocomial pathogen displaying multidrug resistance, Klebsiella pneumoniae, causes respiratory and urinary tract diseases and is a significant threat to immunocompromised individuals (21, 22). Pulmonary infection by the respiratory pathogens C. neoformans and *K. pneumonia* illustrates cross-kingdom similarities of pathogenic mechanisms. For instance, both are facultative intracellular pathogens, produce an antiphagocytic polysaccharide capsule, and interact uniquely with the first line of pulmonary defense, alveolar macrophages (22, 23). This complex scenario of coinfection may influence the host immune response and limit the host’s ability to clear infection, leading to increased mortality rates. Therefore, it is important to improve our understanding of the multidimensional nature of fungal and bacterial infections.

In this study, we use mass spectrometry (MS)-based proteomics to define the host response under single-and dual-infection states of macrophages with C. neoformans and K. pneumoniae to discover critical responses of each biological system. This high-resolution comparative analysis illustrates how protein abundance deviates during a transition from an immediate to established infectious state with exposure to additional microbial stimuli. We reveal global changes upon infection followed by pathogen-specific host response signatures. Additionally, we define regulatory changes within C. neoformans as the fungus adapts to the host environment and stabilizes prior to further disruption in the presence of an established bacterial infection. We validate our findings with host cytokine detection and phenotypic profiling of the fungi throughout the host and bacterial exposures. Overall, our study provides an in-depth analysis of cross-kingdom protein level changes during macrophage infection. This information provides new insight into fungal modulation of the immune response, including stabilization and adaptation of the host and fungi during an established infection, which is disrupted upon prolonged coinfection with a bacterial pathogen. Furthermore, we propose an opportunity to tailor host therapeutic strategies for improved efficacy depending on infection stage and pathogen-specific drivers of disease.

## RESULTS

### Proteome profiling reveals global reprogramming upon coinfection of macrophages.

**FIG 1:**
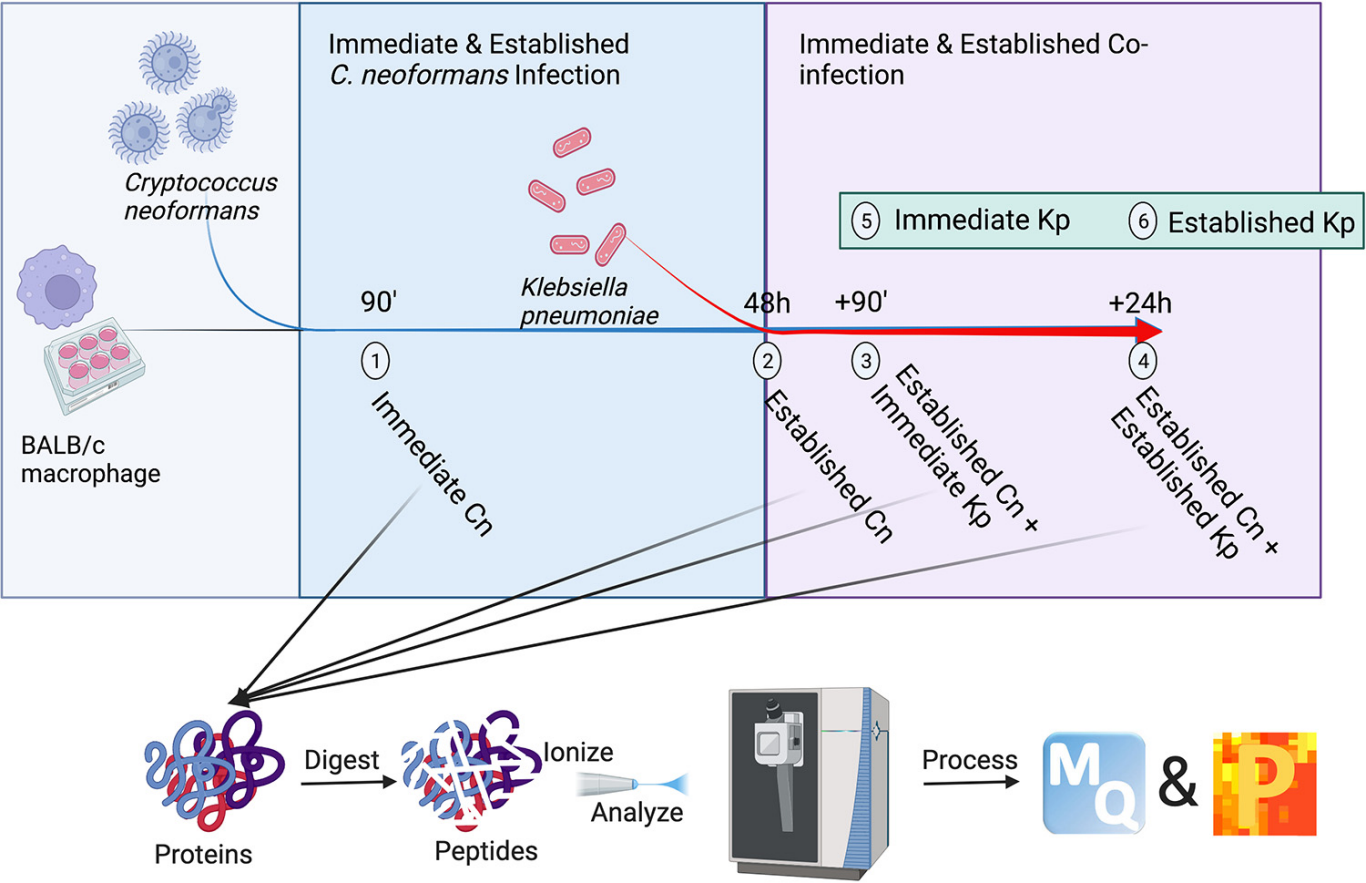
Coinfection workflow for mass spectrometry-based proteomics profiling. BALB/c macrophages were cocultured with C. neoformans H99 (Cn) for 90 min followed by removal of nonphagocytosed cells (i.e., immediate C. neoformans samples) (time point 1). After 48 h, established C. neoformans samples were collected (time point 2), and designated C. neoformans-infected macrophages were cocultured with K. pneumoniae (Kp) for 90 min followed by sample collection (i.e., established C. neoformans plus K. pneumoniae immediate) (time point 3). After 24 h, established C. neoformans plus K. pneumoniae established samples were collected (time point 4). Additionally, K. pneumoniae-only-infected macrophages were collected after immediate (90 min) (time point 5) and established (time point 6) infections. All samples were subjected to our total protein extraction protocol, followed by digestion, measurement on the mass spectrometer, and data processing and analysis using MaxQuant and Perseus. The experiment was performed in biological quadruplicate. The figure generated with Biorender.com.

**FIG 2:**
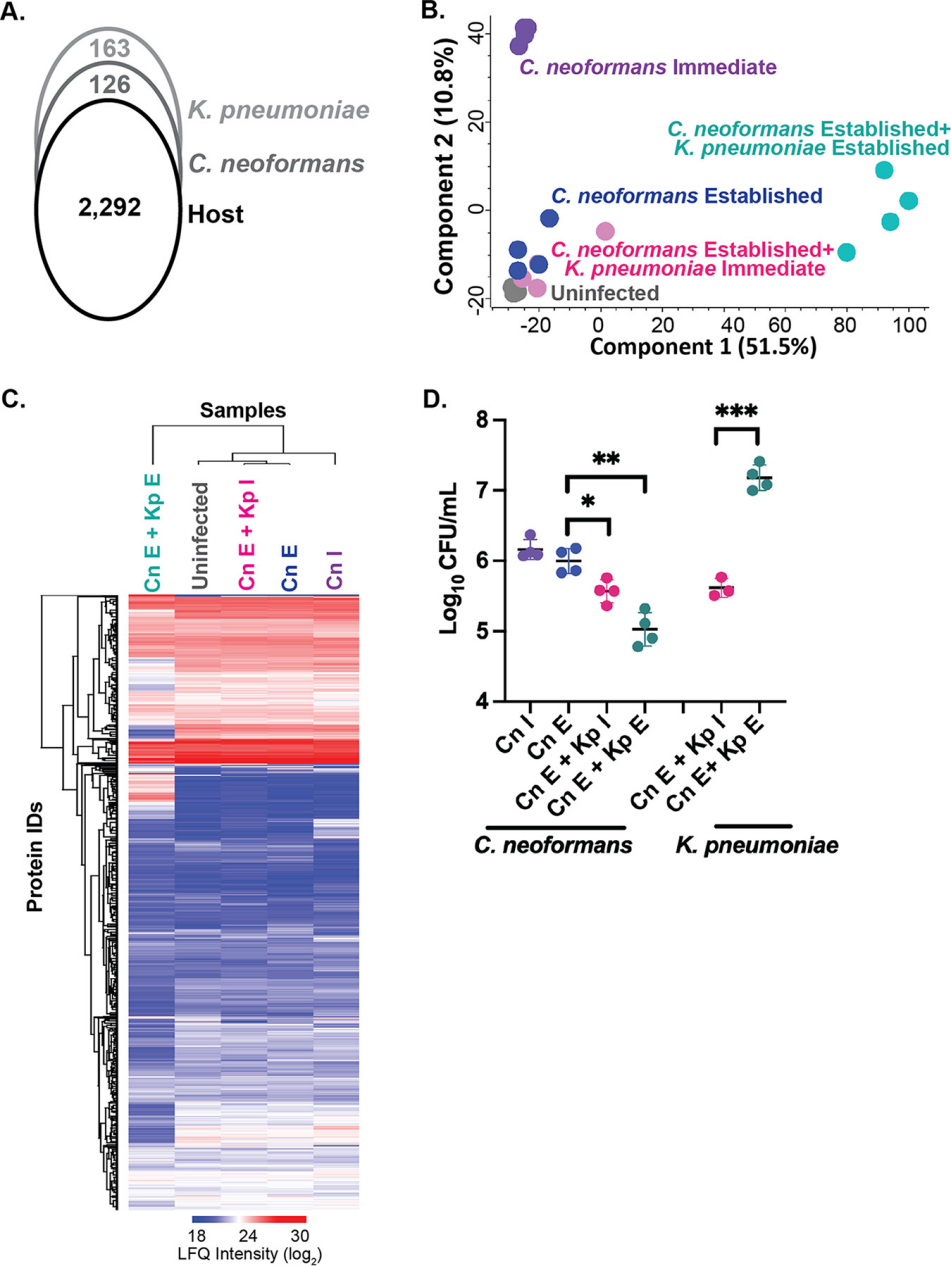
Global proteome response to shifting the macrophage microbiome. (A) Total number of proteins identified for the host (2,292), C. neoformans (128), and K. pneumoniae (167) via proteomics profiling; (B) principal-component analysis of the experiment; (C) heat map of hierarchical clustering by Euclidean distance for the detected proteins across all samples; (D) CFU counts for C. neoformans and K. pneumoniae following infection experiments. Statistical analysis using Student's *t* test: *, *P* < 0.05; **, *P* < 0.001; ***, *P* < 0.0001. The experiment was performed in biological quadruplicate. I, immediate; E, established.

To explore the relationship between immediate and established C. neoformans infection of macrophages and evaluate the impact of coinfection with K. pneumoniae on regulating pathogenic and host responses, we performed quantitative proteomics profiling across a time course of infection (Fig. 1). We define time points representing immediate fungal infection after initial coculture (90 min) and established fungal infection (48 h), which are followed by immediate fungus-bacterium coinfection (90 min) and fungus-bacterium established infection (24 h) for a total experiment duration of approximately 75 h. These time points allow the host time to respond and adapt to the evading pathogen, while also profiling pathogen-specific responses to the changing environment. From a global perspective of infection, we detected 2,292 host proteins, 128 fungal proteins, and 163 bacterial proteins across the samples and time points (Fig. 2A). A principal-component analysis (PCA) defined the largest component of separation was between established co-infection (i.e., C. neoformans established combined with K. pneumoniae established infection) and the other samples, indicating a substantial shift in the proteome upon response to established bacterial colonization (component 1, 51.5%) (Fig. 2B). The second component of separation among the samples was defined by the initial response to immediate fungal infection relative to the other conditions (component 2, 10.8%).

A heat map by Euclidean distance of protein identifications and samples corroborated the PCA results by demonstrating similar protein abundance profiles across the samples, with distinct changes observable in the established coinfection samples (Fig. 2C). We combined our global proteome profile with fungal and bacterial load counts to demonstrate survival and proliferation of the pathogens in the presence of macrophages. Here, we observed a similar number of C. neoformans cells from macrophages upon immediate and established infection, with a progressive reduction in response to bacterial invasion (Fig. 2D). Conversely, for K. pneumoniae, we observed a significant increase over time from immediate to established infection in the presence of C. neoformans within the macrophage. Taken together, these results define a global reprogramming upon coinfection and provide biological support for the survival and proliferation of the pathogens during the infection.

### Host adaptation to established fungal infection is disrupted in the prolonged presence of K. pneumoniae.

**FIG 3:**
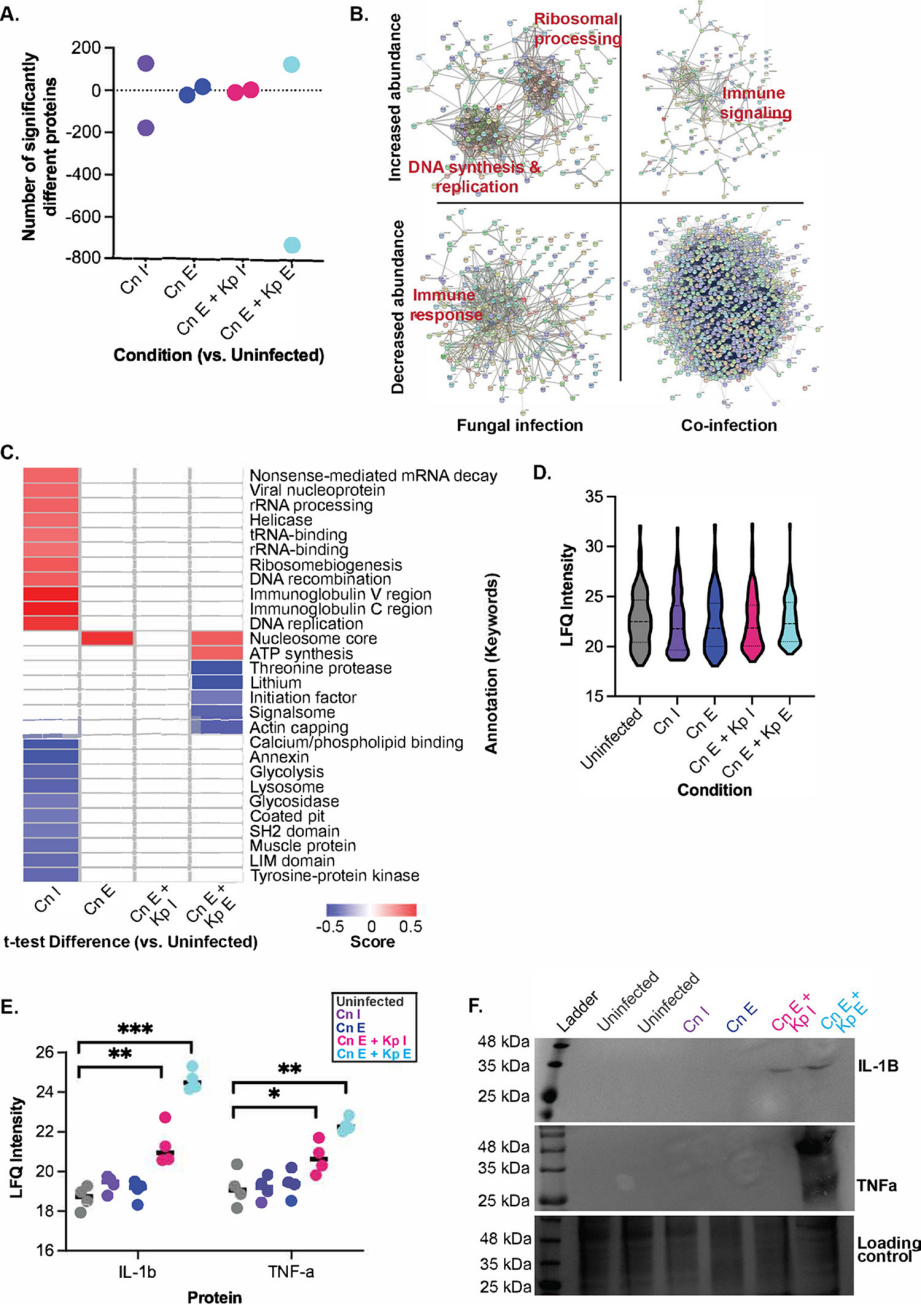
Host-specific response to microbiome reprogramming. (A) Number of significantly different host proteins identified (Student’s *t* test, *P* < 0.05; FDR = 0.05; *S*_0_ = 1) across different conditions; (B) STRING analysis for host proteins with significant changes in abundance across different conditions. Immediate and established infection C. neoformans samples were grouped, and immediate and established infection C. neoformans and K. pneumoniae samples were grouped. Labels represent themes observed within designated clusters based on gene names and protein functions provided in STRING. (C) 1D annotation enrichment for categories of proteins based on keywords. Student's *t* test, *P* < 0.05; FDR = 0.05. (D) Mean LFQ intensity of all immune- and inflammation-associated proteins (as designated by GOBP terms) from the host proteome. (E) Measurement of LFQ intensity of IL-1β and TNF-α from proteome data across conditions. Statistical analysis using Student's *t* test: *, *P* < 0.05; **, *P* < 0.001; ***, *P* < 0.0001. (F) Western blot for 1L-1β and TNF-α across samples and loading control SDS-PAGE. The experiment was performed in biological quadruplicate and technical duplicate.

Given the differences in global proteome profiling, we assessed the impact of coinfection on the host relative to the uninfected macrophage controls. Interestingly, we observed a clear change in proteins that were significantly altered over the time course of infection. For instance, upon immediate fungal infection, the host significantly increased abundance of 127 proteins and decreased abundance of 180 proteins (Fig. 3A; see Table S1 in the supplemental material). Conversely, during established C. neoformans infection, the host appeared to adapt and stabilize its response by significantly altering the production of only 16 proteins (increased) and 24 proteins (decreased). This pattern was still observed in the presence of immediate K. pneumoniae infection, with one protein significantly increased in abundance and 11 proteins significantly reduced. However, upon established bacterial coinfection, a drastic response was observed within the host protein profiles, with 120 proteins significantly increased in production and 729 proteins significantly decreased.

10.1128/mbio.01687-22.1 TABLE S1 List of significantly different host proteins identified during the respective comparisons. Statistical analysis (−log_10_ *P* value reported) by Student’s *t* test: *P* < 0.05, FDR = 0.05, and *S*_0_ = 1. “Difference” represents the difference in LFQ intensities for each sample relative to uninfected macrophage samples. Download Table S1, XLSX file, 0.2 MB. Copyright © 2022 Sukumaran et al. 2022 Sukumaran et al. https://creativecommons.org/licenses/by/4.0/ This content is distributed under the terms of the Creative Commons Attribution 4.0 International license.

Next, we evaluated the impact of these protein-level changes and, in the presence of fungal infection (i.e., immediate and established), observed proteins with increased abundance clustered into two categories via STRING analysis (24): ribosomal processing and DNA synthesis and replication (Fig. 3B). We observed a reduction in abundance of proteins associated with the immune response, indicating that C. neoformans is modulating the host response by suppressing immune function, or if C. neoformans transitions into a dormant phase, the host no longer maintains a defense response (25). In the presence of K. pneumoniae, we observed increased production of immune signaling proteins, suggesting a defense response by the macrophage upon coinfection. We also detected a global reprogramming of proteins with significant reduction in production, which supports the formidable impact that bacterial infection has on macrophages already undergoing remodeling from fungal infection.

A one-dimensional (1D) annotation enrichment (i.e., tests for every annotation term whether the corresponding numerical values have a preference to be systematically larger or smaller than the global distribution of the values for all proteins [26]) was performed based on keywords. In the immediate fungal infection samples, we observed an enrichment of proteins associated with RNA processing and binding, ribosome biogenesis, DNA recombination, helicase, nucleoprotein, and immunoglobulin regions (Fig. 3C). Conversely, a negative enrichment for proteins associated with calcium/phospholipid binding, annexin, glycolysis, lysosome, glycosidase, coated pit, SH2 (Src homology 2) domain, muscle protein, LIM domain, and tyrosine-protein kinase were observed. For the fungal established infection, we observed an enrichment of proteins associated with the nucleosome core and no enriched categories in the presence of immediate K. pneumoniae coinfection. Upon established bacterial infection, we observed an enrichment of proteins within the nucleosome core and ATP synthesis and a negative enrichment in threonine protease, lithium, initiation factor, signalosome, and actin capping. Together, these data support a drastic remodeling upon initial fungal infection, followed by a period of adaptation and stability during established fungal infection and upon initial exposure to K. pneumoniae. This response was followed by another reprogramming upon established bacterial exposure.

Next, we investigated changes in host immune response throughout the duration of infection. We did not observe any significant changes in abundance across the samples of all immune-associated proteins (i.e., proteins were deemed immune associated with protein function by Gene Ontology Biological Processes [GOBP] involved the terms “inflammation” or “immunity”) (Fig. 3D). However, an in-depth look at immune-associated proteins with significant changes in abundance during coinfection revealed interleukin-1β (IL-1β) and tumor necrosis factor alpha (TNF-α) showed significant increases in production in the presence of bacterial cells (Fig. 3E). These results were confirmed by Western blotting (Fig. 3F). Overall, remodeling of the host response is extensive and dynamic over the course of infection, and specific immune responses tailored to bacterial coinfection were observed.

### C. neoformans initiates and responds to infectious states while permitting adaptation during a stable established infection.

**FIG 4:**
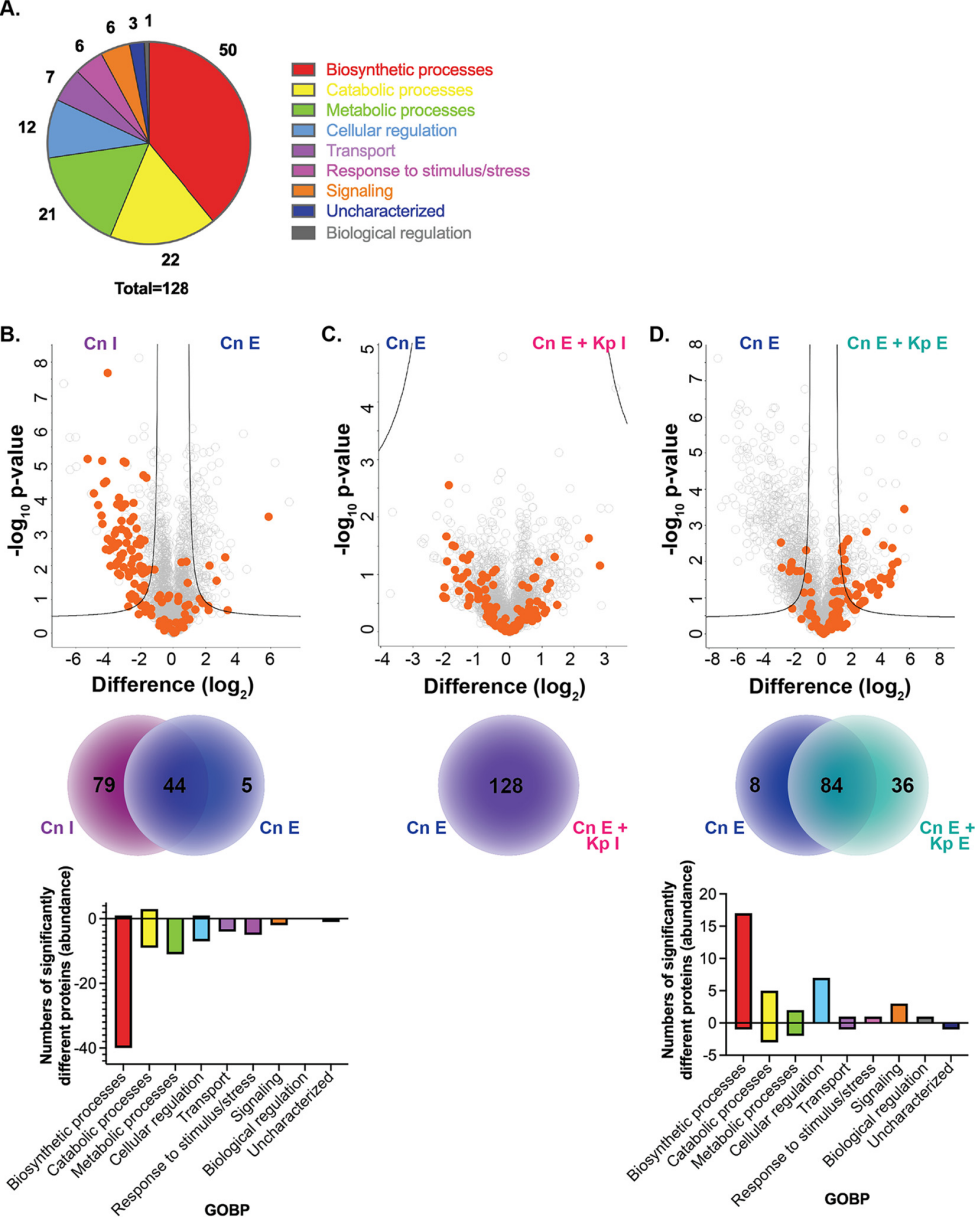
Fungal proteome signatures upon invasion, adaptation, and disruption. (A) Distribution of GOBP terms for identified fungal proteins. (B) Volcano plot for comparison of fungal proteins between immediate and established C. neoformans infection. The number of significantly different proteins identified under each condition (Venn diagram) and distribution of GOBP terms between fungal proteins with significant increases or decreases in abundance upon infection are shown. (C) Volcano plot for comparison of fungal proteins between established C. neoformans infection and coinfection (established C. neoformans plus immediate K. pneumoniae infection). The number of proteins identified under each condition is shown (Venn diagram). (D) Volcano plot for comparison of fungal proteins established C. neoformans infection and established coinfection (established C. neoformans plus established K. pneumoniae infection). The number of significantly different proteins identified under each condition (Venn diagram) and distribution of GOBP terms between fungal proteins with significant increases or decreases in abundance upon infection are shown. Volcano plots: Student’s *t* test, *P* < 0.05; FDR = 0.05; *S*_0_ = 1. Orange denotes C. neoformans proteins. The experiment was performed in biological quadruplicate.

To tease apart the impact and response of C. neoformans to coculture with macrophages, followed by coinfection with K. pneumoniae, we defined fungus-specific adaptations over time. Our analysis identified 128 fungal proteins across the samples, and an assessment of protein functions based on GOBP showed many proteins associated with biosynthetic (27), catabolic (22), and metabolic processes (21), followed by cellular regulation (12), transport (7), response to stimulus/stress (6), signaling, (6) and biological regulation (1), as well as uncharacterized roles (3) (Fig. 4A). Volcano plot comparisons emphasize changes in the fungal proteomes aligned with the remodeling changes observed in the host. For instance, a comparison of fungal proteins between immediate and established infection revealed a significant increase in abundance of 79 proteins during the immediate phase compared to five proteins with higher abundance under established conditions (Fig. 4B; Table S2). Many of the proteins identified with higher abundance during the immediate response had roles in biosynthetic, catabolic, and metabolic processes, whereas during established infection, catabolic processes and cellular regulation were impacted. After alignment with the observed host adaptation and stabilization during established fungal infection in the absence and presence of K. pneumoniae, we did not obverse any significant differences in fungal protein production (Fig. 4C). Conversely, during established coinfection conditions, C. neoformans responded to the presence and proliferation of K. pneumoniae by increasing production of 36 proteins associated with biosynthetic, catabolic, and metabolic processes, as well as cellular regulation and signaling (Fig. 4D; Table S3). This global reprogramming after a state of dormancy indicates that the fungus is still capable of responding to changes in its environment, either directly initiated by the presence of the bacteria or upon alteration of the host to the invading pathogen.

10.1128/mbio.01687-22.2 TABLE S2 List of significantly different fungal proteins identified during C. neoformans established versus immediate infection. Statistical analysis (−log_10_ *P* value reported): Student’s *t* test, *P* < 0.05; FDR = 0.05; *S*_0_ = 1. “Difference” represents the difference in LFQ intensities for C. neoformans established versus immediate infection samples. Download Table S2, XLSX file, 0.02 MB. Copyright © 2022 Sukumaran et al. 2022 Sukumaran et al. https://creativecommons.org/licenses/by/4.0/ This content is distributed under the terms of the Creative Commons Attribution 4.0 International license.

10.1128/mbio.01687-22.3 TABLE S3 List of significantly different fungal proteins identified during established coinfection versus C. neoformans established infection. Statistical analysis (−log_10_ *P* value reported): Student’s *t* test, *P* < 0.05; FDR = 0.05; *S*_0_ = 1. “Difference” represents the difference in LFQ intensities for established coinfection versus C. neoformans established infection samples. Download Table S3, XLSX file, 0.01 MB. Copyright © 2022 Sukumaran et al. 2022 Sukumaran et al. https://creativecommons.org/licenses/by/4.0/ This content is distributed under the terms of the Creative Commons Attribution 4.0 International license.

### C. neoformans restricts catalase production in the presence of K. pneumoniae.

**FIG 5:**
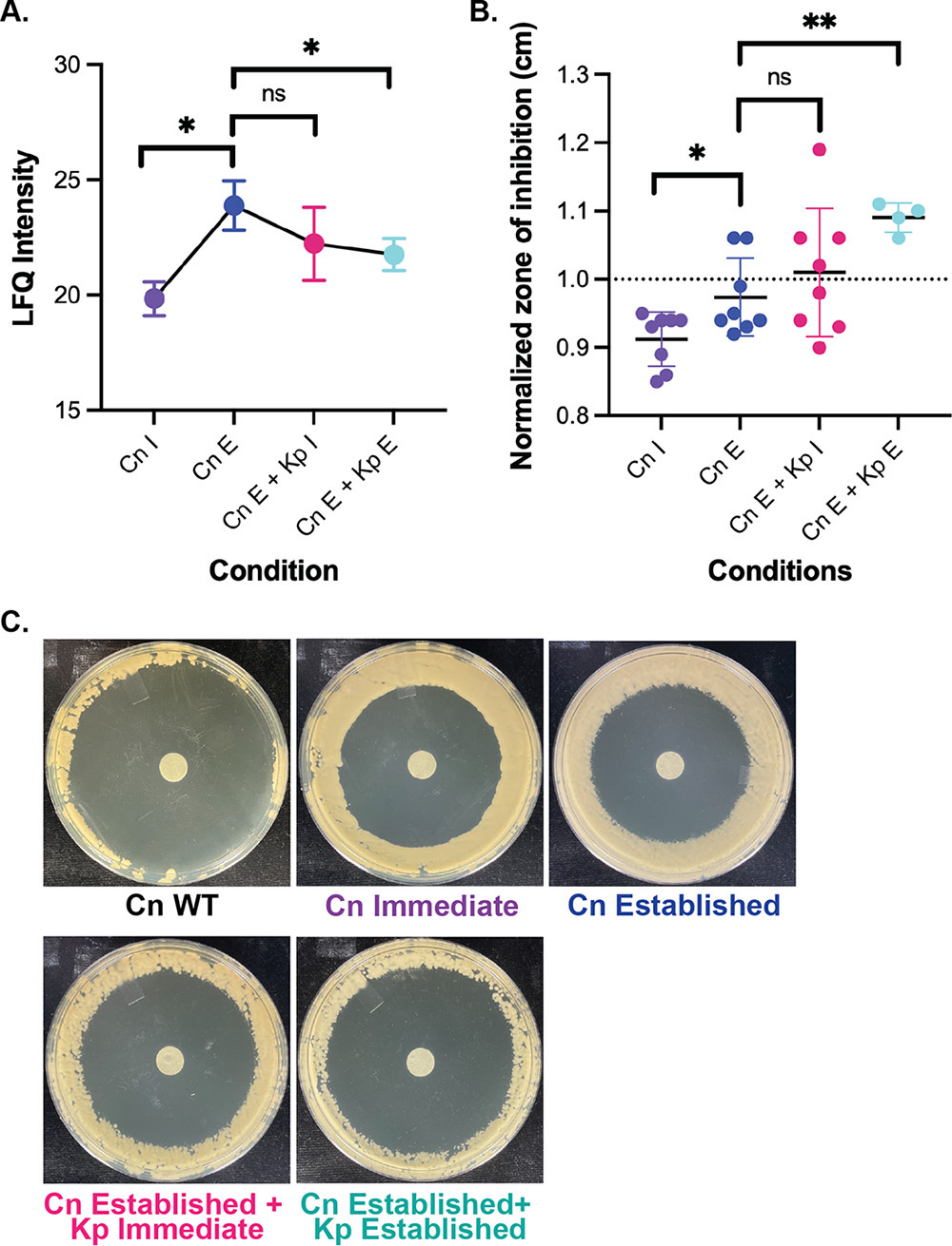
Fungal catalase production during coinfection. (A) Mean LFQ intensity for two C. neoformans catalase proteins identified by proteomic profiling; (B) normalized zone of inhibition measurements by H_2_O_2_ plate assays. Data were normalized to the C. neoformans WT. (C) H_2_O_2_ plate assays across conditions. Data were normalized to C. neoformans WT. Statistical analysis using Student's *t* test: *, *P* < 0.05; **, *P* < 0.001. Images were taken 48 h after incubation at 37°C with 10% H_2_O_2_. The experiment was performed in biological quadruplicate and technical duplicate.

Of the cryptococcal proteins produced during infection of macrophages, we observed two catalases (i.e., CAT3 and CAT1) with known roles in antioxidant defense toward the host (27). A closer look at these proteins revealed a profile of high production during established C. neoformans infection of the macrophage, followed by a significant reduction in catalase abundance under established bacterial coinfection (Fig. 5A). For C. neoformans immediate infection samples, only one catalase was detected in the proteome (i.e., CNAG_04891) with a significantly lower abundance than the fungal established infection samples. We did not observe a significant change in catalase protein profiles between the established fungal and immediate bacterial co-infections. Next, we evaluated the levels of catalase production across the established infection samples by measuring a zone of inhibition in the presence of reactive oxygen species (e.g., H_2_O_2_) via phenotypic profiling. We observed a significant increase in the zone of inhibition between the established fungal and established coinfection samples, indicating lower catalase production during prolonged coinfection (Fig. 5B). Interestingly, we observed the highest level of catalase production in the C. neoformans immediate infection samples (denoted by the smallest zone of inhibition), which did not correspond with the single detectable catalase protein profile. Again, we did not observe a significant change in catalase production between the established fungal and immediate bacterial infections. The results for catalase protein profiles during established co-infections were corroborated by plate assays; however, an inverse protein profile compared to the zone of inhibition was observed for the C. neoformans immediate samples (Fig. 5C). Taken together, these data support an increased production of catalase under established fungal infections, which decreases under prolonged exposure to bacterial coinfection.

### C. neoformans responds to host invasion with rapid melanin production that decreases with reduced host ROS production.

**FIG 6:**
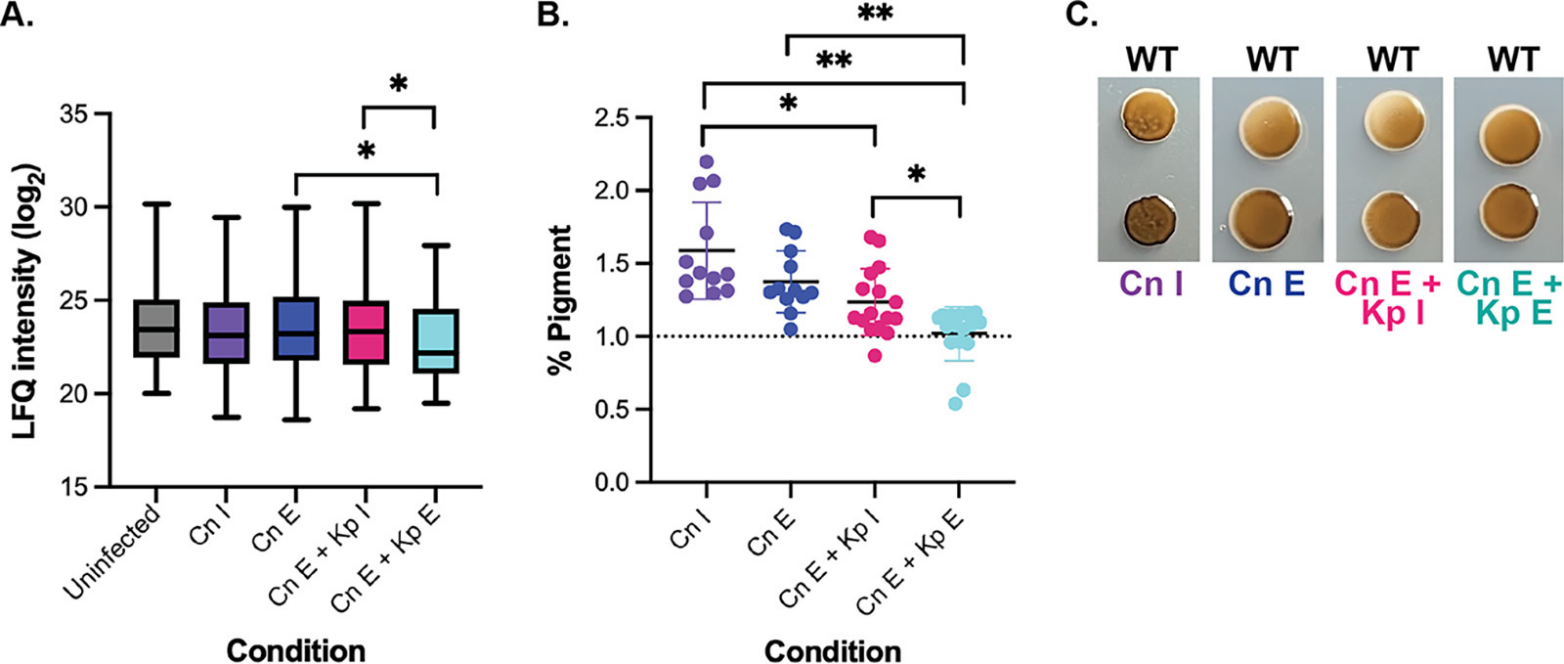
Host ROS response and fungal melanin production are interconnected. (A) LFQ intensity of host proteins designated by GOBP for regulation and metabolism of reactive oxygen species across the tested conditions; (B) normalized percentage of melanin (pigment) produced by C. neoformans colonies collected from macrophages following infection. Data were normalized to the C. neoformans WT. (C) l-DOPA plate assay to visualize melanin production by C. neoformans. Statistical analysis using Student's *t* test: *, *P* < 0.05; **, *P* < 0.001. Images were taken 96 h after incubation at 37°C. The experiment was performed in biological quadruplicate and technical duplicate.

In response to environmental stressors, including the production of reactive oxygen species (ROS) by the host, C. neoformans produces melanin, which is associated with virulence, cell wall stability, and protection from antimicrobial hydroxyl radicals (28, 29). By focusing on the production of host proteins with roles in the regulation and metabolism of ROS, we identified 42 proteins within our data set. Assessment of protein abundance defined a stable ROS production in the uninfected, C. neoformans immediate infection, and established infection samples, as well as the K. pneumoniae immediate co-infection samples, whereas a significant reduction in melanin production was observed during established fungal and bacterial coinfection (Fig. 6A). Upon quantification of melanin production by C. neoformans on l-3,4-dihydroxyphenylalanine (l-DOPA) plates following the coinfection experiments, the highest level was measured in the fungal immediate samples, followed by the fungal established samples and the bacterial immediate samples (Fig. 6B). Similarly, we observed a significant reduction in melanin in the established coinfection samples compared to the earlier time points, with production comparable to that of wild-type (WT) C. neoformans (i.e., baseline). These observations were supported by visual inspection of the l-DOPA plates (Fig. 6C). These data support a tailored response of C. neoformans through the production of melanin upon infection of macrophages; a baseline level of melanin corresponds with a reduction in host ROS.

### K. pneumoniae proteome response amplifies during established coinfection.

**FIG 7:**
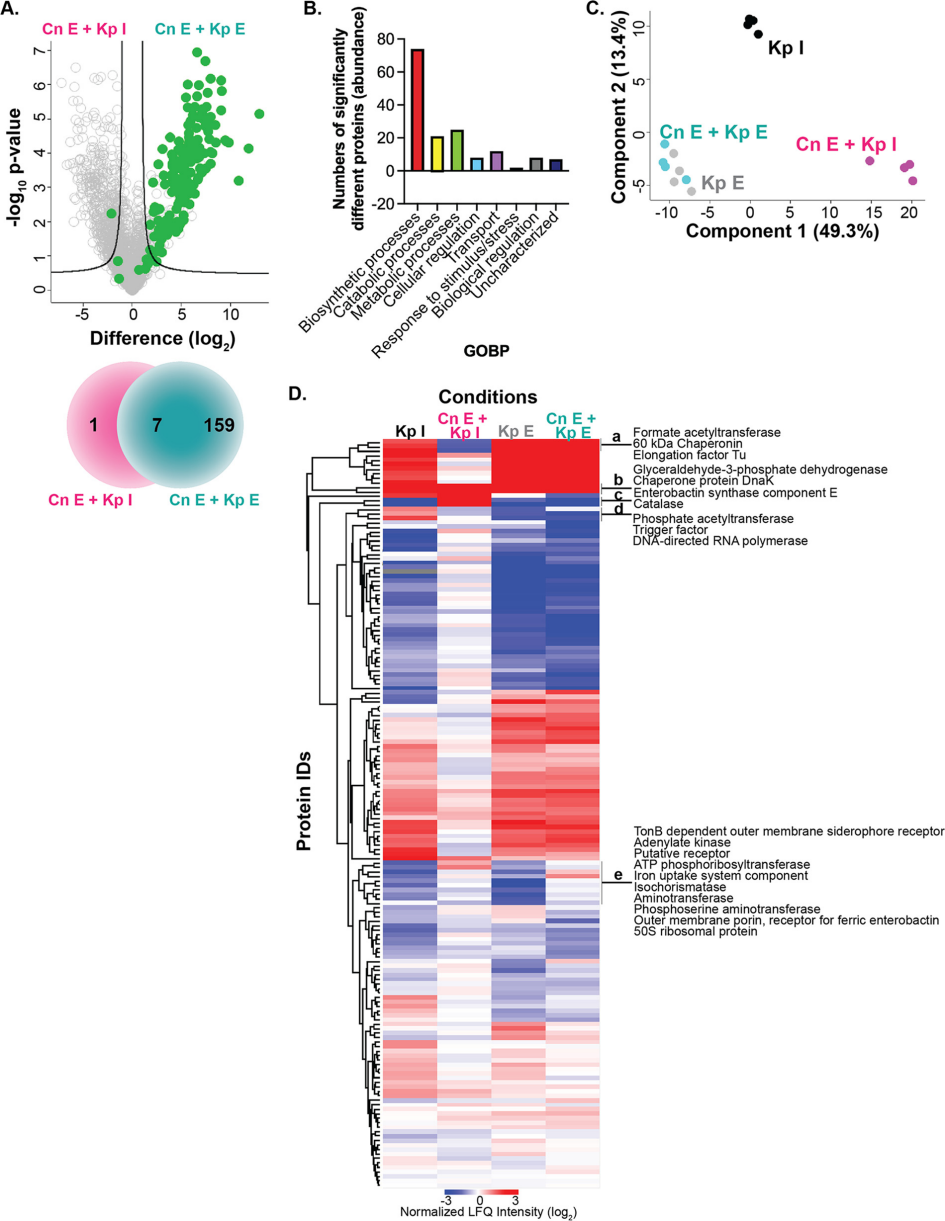
Enhanced bacterial protein response upon established coinfection. (A) Volcano plot for comparison of bacterial proteins between established C. neoformans plus immediate K. pneumoniae coinfection and established C. neoformans plus established K. pneumoniae coinfection. The Venn diagram depicts the number of significantly different proteins identified under each condition. (B) Distribution of GOBP terms between bacterial proteins with significant increases or decreases in abundance upon coinfection. (C) PCA plot of the experiment. (D) Heat map of normalized LFQ intensities by Euclidean distance for K. pneumoniae proteins identified under the specified conditions. Volcano plots: Student’s *t* test, *P* < 0.05; FDR = 0.05; *S*_0_ = 1. Green denotes K. pneumoniae proteins. The experiment was performed in biological quadruplicate. Letters a to e designate a protein cluster for reference as described within the main text.

We detected 167 bacterial proteins during our proteome profiling of macrophage coinfection at immediate (90 min) and established (24 h) time points in the presence of C. neoformans. We performed corresponding control experiments with macrophages infected with K. pneumoniae following 90 min and 24 h of incubation to distinguish bacterium-fungus interaction proteome signatures. K. pneumoniae was able to survive and replicate within macrophages during coinfection, and we observed a significant increase in abundance of 159 bacterial proteins during the established infection stage, whereas only one protein (a catalase) was significantly higher during immediate bacterial infection (Fig. 7A; Table S4). Seven proteins remained consistent during immediate versus established coinfection, and a substantial remodeling of proteins was observed at the later time point. Defining represented categories by GOBP, we observed a substantial number of proteins associated with biosynthetic, catabolic, and metabolic processes with increased abundance during established coinfection, as well as proteins involved in cellular regulation, transport, response to stimulus/stress, or biological regulation or which were uncharacterized (Fig. 7B). Notably, we observed 37 bacterial proteins with defined roles in metal ion binding and transport, including outer membrane proteins (e.g., OmpX and OmpA), iron regulators (e.g., IroN and ferric enterobactin protein [FepA]), secretion and export (e.g., SecB), and virulence (e.g., superoxide dismutase [SodB]) with higher abundance during established infection. These data support reprogramming of the bacterium for survival within the nutrient-limited and hostile environment of the host.

10.1128/mbio.01687-22.4 TABLE S4 List of significantly different bacterial proteins identified during established coinfection versus C. neoformans established and K. pneumoniae immediate infection. Statistical analysis (−log_10_ *P* value reported): Student’s *t* test, *P* < 0.05; FDR = 0.05; *S*_0_ = 1. “Difference” represents the difference in LFQ intensities for established coinfection versus C. neoformans established and K. pneumoniae immediate infection samples. Download Table S4, XLSX file, 0.02 MB. Copyright © 2022 Sukumaran et al. 2022 Sukumaran et al. https://creativecommons.org/licenses/by/4.0/ This content is distributed under the terms of the Creative Commons Attribution 4.0 International license.

We then incorporated data from *K. pneumonia*-only macrophage infections following immediate (90 min) and established (24 h) infection models and compared the normalized protein profiles to the coinfection models. By PCA, the largest distinction within the data set was between immediate versus established infections (i.e., component 1, 49.3%), with the second component distinguishing immediate K. pneumoniae-only infection (i.e., component 2, 13.4%) (Fig. 7C). Using a heat map based on hierarchical clustering by Euclidean distance of protein IDs, we defined unique signatures of protein production dependent on the infection model (i.e., distinction between immediate versus established and single infection versus coinfection) (Fig. 7D). Here, we observed five signatures outlining bacterium-specific responses to the host condition. For instance, we defined a consistent increase in abundance of three K. pneumoniae proteins (i.e., formate acetyltransferase, 60-kDa chaperonin, and elongation factor Tu) across all conditions, except during immediate infection of a C. neoformans established culture, designated “a.” Conversely, we observed an increase in abundance of virulence-associated bacterial proteins (i.e., enterobactin synthase component E, catalase) under the same condition, designated “c.” These data support the activation of K. pneumoniae virulence factors tailored against the present fungal pathogen, as we reported above. Additionally, during immediate bacterial infection, we detected an increase in abundance of three proteins (i.e., phosphate acetyltransferase, trigger factor, and DNA-directed RNA polymerase), designated “d.” This observation supports a role for the proteins during initial bacterial infection of the macrophage, which is not maintained as infection progresses or in a preprimed (i.e., infected) macrophage. Notably, we observed a consistent increase in abundance of two proteins (i.e., Glyceraldehyde-3-phosphate dehydrogenase [GAPDH], chaperone protein DnaK) across all conditions, which supports an infection- and time-independent response, designated “b.” We also observed a unique signature between the established single-infection versus coinfection models, with reduced production of 10 bacterial proteins in the absence of C. neoformans, designated “e.” Taken together, these control experiments define fungus-driven bacterial responses during coinfection of macrophages and reveal opportunities for targeted disruption and treatment of K. pneumoniae upon coinfections.

## DISCUSSION

In this study, we used quantitative proteomics to explore the dynamic relationship between macrophages and pathogens during immediate and established single-infection and coinfection models. We provide novel insight into global remodeling of the proteome upon exposure to the pathogens, as well as host-specific immune response signatures tailored to the specific pathogen. We highlight the fungal proteins detected upon infection and observe an adaptation and stabilization of both the host and C. neoformans during established infection, suggesting that the biological systems reach homeostasis following immediate infection. This “calm before the storm” is disrupted in the prolonged presence of K. pneumoniae as the bacterium replicates and reprograms its proteome to respond to host defenses. Additionally, we support our proteome findings with phenotypic assays quantifying catalase and melanin production by C. neoformans over the duration of the infection models. Overall, our study deepens our understanding of each biological system and the interplay during complex diseases, as well as provides new insight into the impact of microbiome modulation during disease.

The most well-characterized kingdom of the microbiota constitutes the “bacteriome”; however, this complex ecosystem harbors a diverse profile of underrepresented members, including archaea, viruses, and fungi (1). In recent years, the spotlight has shifted to the neglected but influential fungal components comprising the “mycobiota” (1, 10). This increased attention has recognized the role of the mycobiota in multiple human mucosal sites, including its involvement in pathological conditions, such as inflammatory bowel disease (30). Furthermore, the human respiratory tract offers a port of entry for transient and colonizing microorganisms due to the high exposure to airborne particles. Thus, depending on the host's immune status and characteristics of colonizing microbe, the oral and pulmonary microbiome will have a distinct profile unique to each individual (3). We observed a remodeling of the host in the presence of the fungal pathogen, along with an adaptation and return to homeostasis during established fungal infection. This observation aligns well with the ability of C. neoformans to remain within an infected host in a dormant state for years, going undetected by the immune system, unless a disruption to this homeostasis is encountered (13). Such disruptions may include a shift to an immunocompromised status for the host or the presence of a coinfection pathogen, as we observe in this study (13, 31, 32).

We provide the first comprehensive proteomic profiling of the interplay during cross-kingdom coinfection and have begun to tease apart fungal responses tailored to the host compared to responses elicited by the bacterium. For instance, C. neoformans modulates the production of catalase and melanin during infection with bursts of production upon initial interactions with macrophages but dissipates levels of these two well-characterized virulence factors over time. We defined reduced production of catalase in the C. neoformans immediate samples using proteomics with elevated levels of catalase in our *in vitro* assays. Previous reports stated the role of CAT1 in mediating *in vitro* catalase activity (27, 33), and given our detection of a single catalase at the protein level, we may not capture the comprehensive role of catalases in these assays. For example, C. neoformans catalases may contribute to the oxidate stress response of the host in a cooperative manner and not independently, which would influence our findings. Such findings could be elaborated upon with further assessment of catalase-deficient fungal strains and their impact in responding to ROS production by the host at the protein level.

Upon coinfection with established K. pneumoniae, we observed a significant increase in production of a signaling cascade regulating enzyme, STE protein kinase (CNAG_03290). This enzyme has been linked to increased melanin production upon gene deletion (34). These earlier findings further support our observation of decreased melanin production over time in response to lower host ROS and possible pigment regulation by CNAG_03290. Previous studies demonstrated the regulation of fungal virulence determinants in response to bacterial presence. For instance, in the presence of Klebsiella aerogenes, an increase in cryptococcal melanin production was observed (18). Although we observed a reduction in melanin over time in our assays, conflicting with the previous report, we acknowledge that our use of a hypervirulent K. pneumoniae strain may alter the response by C. neoformans under established infection, as well as the presence of host cells, and the consideration of ROS in regulating melanin production may also support this deviation. Moreover, previous findings for Aspergillus spp., in which the presence of K. pneumoniae inhibited spore germination and hypha formation and decreased biofilm formation (35), align with our observed reduction in fungal virulence factor production in the presence of dual infection over time. Together, our data support adaptation of the fungi to the host environment and highlight limited effectiveness in the presence of coinvading K. pneumoniae. However, a shift toward increased production of fatty acid synthase (e.g., Fas2) during established bacterial coinfection may provide a window of opportunity to disarm the fungi through inhibition of these proteins (36) and prevent an active infection within the host.

The practice of overprescribing antibiotics may alter the host’s ability to maintain microbial homeostasis and provides an opportunity for the fungal-bacterial abundance to shift and generate an environment prone to the establishment of infectious diseases (37, 38). C. neoformans interaction with host immune cells may lead to extracellular survival or intracellular replication within the phagolysosome (23). Whereas K. pneumoniae promotes an intracellular replicative niche by inhibiting phagosomal maturation (39), our study supports intracellular survival of C. neoformans through fungal plate counts in the immediate and established fungal infection samples; however, in the presence of K. pneumoniae, we observed a rapid and significant reduction in fungal cell survival possibly connected to increased competition for resources within the nutrient-limited environment of the host. Conversely, our data support the formation of an intracellular replication niche for K. pneumoniae, which rapidly increased over 10-fold in cell counts between immediate and established infections. These data support a minimal impact of fungal defense response against the bacterium and a robust response of the bacterium toward host defenses, such as the production of ROS.

We also observed increased production profiles for bacterial proteins associated with nutrient acquisition, virulence, and transport that promote its survival. For instance, a connection between iron availability and the production of the export protein SecB has been previously demonstrated in K. pneumoniae (40), as well as outer membrane proteins associated with promoting bacterial virulence (OmpX) (41). Moreover, the outer membrane protein OmpA mediates adhesion and/or invasion of eukaryotic cells (42, 43) and confers resistance to antimicrobial peptides produced by the host, which may support bacterial survival and proliferation (44). Also directly supported by our findings is the overstimulation of host Toll-like receptors and the downstream inflammatory responses of TNF-α and IL-1β in the presence of lipid A, an important component of the K. pneumoniae lipopolysaccharide, which regulates immune response (45). Furthermore, our observation of fungus-driven bacterial responses to infection defined a role for enhanced nutrient acquisition (i.e., TonB-dependent outer membrane siderophore receptor, a putative receptor, the iron uptake system component isochorismatase, and outer membrane porin) in the presence of C. neoformans. These findings were anticipated given the competition for nutrients by K. pneumoniae and C. neoformans within nutrient-limited environments (40, 46, 47). Furthermore, we observed regulation of signaling networks (i.e., adenylate kinase), metabolism (i.e., ATP phosphoribosyltransferase, aminotransferase, phosphoserine aminotransferase), and translation (i.e., 50S ribosomal protein), which support global changes to bacterial protein production driven by the presence of an established fungal infection.

### Conclusion.

Cross-kingdom infections regulate many aspects of the disease, including efficiency and efficacy of the host response and the relationship between the invading pathogens. The status of infection, whether it be an immediate, initial infection that causes an effective innate response or a latent, established infection that promotes adaptation of the host and fungi for survivability, changes over time. Additionally, the disruption of a newfound homeostasis during established fungal infection by an invading bacterial pathogen leads to an amplified immune response and scavenging of nutrients by the pathogen. Critically, our observations define new relationships during dual infection of macrophages and highlight opportunities to target pathogenic proteins produced during specific times and stages of infection, which may improve our ability to combat diseases, especially with the ever-increasing devastation of antimicrobial resistance.

## MATERIALS AND METHODS

### Fungal strains, growth conditions, and media.

Cryptococcus neoformans variety *grubii* wild-type (WT) strain H99 (serotype A) was used for all analyses. C. neoformans was maintained on yeast extract-potato dextrose (YPD) agar plates (2% dextrose, 2% peptone, 1% yeast extract, 1.5% agar) at 30°C unless otherwise stated.

### Bacterial strains, growth conditions, and medium preparation.

The K. pneumoniae WT (K52 serotype) was maintained on lysogeny broth (LB) agar plates (10% tryptone, 0.5% yeast extract, 1% NaCl, 1.5% agar). For macrophage infections and *in vitro* cultures, K. pneumoniae was grown overnight at 37°C in LB, subcultured into LB, and grown to mid-log phase.

### Macrophage infection.

BALB/c WT immortalized macrophages (generously provided by Felix Meissner, Max Planck Institute of Biochemistry, Germany) were maintained at 37°C in 5% CO_2_ in Dulbecco’s modified Eagle’s medium (DMEM) supplemented with 10% heat-inactivated fetal bovine serum (FBS), 2 mM Glutamax, 1% sodium pyruvate, 1% l-glutamine, and 5% penicillin-streptomycin (pen-strep). Macrophages were seeded in 6-well plates at 0.3 × 10^6^ cells/well and grown for 48 h until 70 to 80% confluence was reached (i.e., 1.2 × 10^6^ cells/mL).

Infection was performed as we previously described (48). Briefly, C. neoformans strains were grown to mid-log phase in YPD at 37°C, collected at 1,500 × *g* for 10 min, washed twice in phosphate-buffered saline (PBS), and resuspended in DMEM without pen/strep. Fungal cells were opsonized with antiglucuronoxylomannan (GXM) monoclonal antibody (MAb) 18B7 (1 μg/10^6^ fungal cells) for 1 h at 37°C and 5% CO_2_. Macrophages were infected at a multiplicity of infection (MOI) of 5:1 (cryptococcal to macrophage cells) for 90 min at 37°C at 5% CO_2_. Following coculture, cells were washed with PBS to remove nonphagocytosed fungal cells, and fresh DMEM without pen/strep supplemented with 20 μg/mL fluconazole was added for the remainder of the assay.

For coinfection assays, K. pneumoniae cells were cultured to mid-log phase, centrifuged at 3,500 × *g* for 10 min, washed twice in PBS, and resuspended in complete DMEM without pen/strep supplemented with 20 μg/mL fluconazole. Macrophages were infected at an MOI of 100:1 for 90 min at 37°C, 5% CO_2_. Subsequently, cells were washed with PBS and incubated at 37°C with 5% CO_2_ in growth medium containing 300 μg/mL gentamicin for 90 min. Medium was replaced to decrease the gentamicin concentration to 100 μg/mL and either collected at 0 h (i.e., immediate) or incubated for 24 h (i.e., established).

Samples were collected at the following time points: 1, initial C. neoformans infection (i.e., 90 min, immediate cryptococcal infection); 2, 48 h after initial cryptococcal infection (i.e., established cryptococcal infection); 3, coinfection following initial K. pneumoniae infection (i.e., 90 min, established cryptococcal infection plus immediate K. pneumoniae infection); 4, coinfection 24 h following K. pneumoniae infection (i.e., established cryptococcal infection plus established K. pneumoniae infection); 5, immediate K. pneumoniae infection (i.e., K. pneumoniae and macrophages after 90 min); and 6, established K. pneumoniae infection (i.e., K. pneumoniae and macrophages after 24 h). Control samples consisting of uninfected macrophages were maintained in DMEM without pen/strep with 20 μg/mL fluconazole to correspond with the development of an established cryptococcal infection. Experiments performed in biological quadruplicate.

### Proteomics sample preparation.

Samples were prepared as we previously described (49). Briefly, samples were collected in 100 mM Tris-HCl (pH 8.5) containing a protease inhibitor cocktail tablet (Sigma-Aldrich), followed by addition of sodium dodecyl sulfate (SDS [2% final concentration]) and probe sonication (Thermo Fisher Scientific). Dithiothreitol (DTT [10 mM final concentration]) and iodoacetamide (IAA [5.5 mM final concentration]) were added, followed by acetone precipitation overnight at −20°C. Samples were collected, washed, and resuspended in 8 M urea–40 mM HEPES for protein concentration measurement using a bovine serum albumin (BSA) tryptophan assay (50). Samples were diluted in 50 mM ammonium bicarbonate, normalized to 50 μg, and digested with LysC-trypsin (Promega [protein/enzyme ratio, 50:1]). To stop digestion, 10% (vol/vol) trifluoroacetic acid (TFA) was added, and peptides were purified using C_18_ stop-and-go-extraction tips (StageTips) (51).

### LC-MS/MS.

For liquid chromatography-tandem mass spectrometry (LC-MS/MS), digested peptides were resuspended in buffer A (0.1% formic acid) and analyzed on a Orbitrap Exploris 240 hybrid quadrupole-orbitrap mass spectrometer (Thermo Fisher Scientific) coupled to an Easy-nLC 1200 high-performance liquid chromatography device (Thermo Fisher Scientific). Samples were loaded onto an in-line 75-μm by 50-cm PepMap RSLC EASY-Spray column filled with 2-μm C_18_ reverse-phase silica beads (Thermo Fisher Scientific). Separated peptides were electrosprayed into the mass spectrometer with a linear gradient of 3% to 20% buffer B (80% acetonitrile, 0.5% acetic acid) over a 3-h gradient, followed by a wash with 100% buffer B with a 250-nL/min flow rate. The mass spectrometer switched between one full scan and MS/MS scans of abundant peaks. Full scans (*m*/*z* 400 to 2,000) were acquired in the Orbitrap mass analyzer with a resolution of 120,000 at 200 *m*/*z* 200.

### Data processing.

Analysis of mass spectrometry raw data files was performed using MaxQuant software (version 1.6.0.26) (52). The search was completed using the incorporated Andromeda search engine against the reference C. neoformans var. *grubii* serotype A (strain H99/ATCC 208821) proteome (7,430 sequences; 31 January 2020) and K. pneumoniae K52 serotype (5,126 sequences; 21 May 2020), and Mus musculus (55,462 sequences; 1 June 2020) from Uniprot (53). The following parameters were included: trypsin enzyme specificity with a maximum of two missed cleavages, a minimum peptide length of seven amino acids, fixed modifications, including carbamidomethylation of cysteine, and variable modifications, including, methionine oxidation and N-acetylation of proteins and split by taxonomic ID. Peptide spectral matches were filtered using a target-decoy approach at a false-discovery (FDR) of 1% with a minimum of two peptides required for protein identification. Relative label-free quantification (LFQ) and match between runs were enabled; the MaxLFQ algorithm used a minimum ratio count of 1 (54).

### Bioinformatics.

Statistical analysis and data visualization of the proteomics data were performed using Perseus (version 1.6.2.2) (55). Data were prepared by filtering for reverse database matches, contaminants, and proteins only identified by site, followed by log_2_ transformation of LFQ intensities. Filtering for valid values (three of four replicates in at least one group) was performed, and missing values were imputed from the normal distribution (width, 0.3; downshift, 1.8 standard deviations). Significant differences were evaluated by a Student’s *t* test (*P* ≤ 0.05) with multiple-hypothesis testing correction using the Benjamini-Hochberg (56) FDR cutoff at 0.05 with *S*_0_ = 1. STRING analysis was performed as described at https://string-db.org. The 1D annotation enrichment was performed as previously described (26).

### Microbial cell count.

To determine survival of the two microbial species under the various conditions, at the time of collection, an aliquot was serially diluted in PBS. Undiluted (100 μL) and diluted (ranging from 10^2^ to 10^5^) cultures were plated on LB supplemented with 30 μg/mL fluconazole to isolate K. pneumoniae cells or YPD supplemented with 32 μg/mL chloramphenicol to isolate C. neoformans cells. Plates were incubated at 37°C for 1 or 2 days, respectively, and CFU were enumerated. Experiments were performed in biological quadruplicate and technical duplicate.

### Western blot.

Aliquots of undigested protein extract from the proteomic sample preparation highlighted above were separated by SDS-PAGE and transferred to a polyvinylidene fluoride (PVDF) membrane using a transfer apparatus according to the manufacturer’s instructions (Bio-Rad). Membranes were blocked for 2 h in 5% nonfat skim milk in TBS (50 mM Tris, 150 mM NaCl [pH 7.5]) at room temperature (RT), washed five times with TBST (1× TBS, 0.05% Tween 20) and incubated with either IL-1β polyclonal antibody (Thermo Fisher Scientific) or TNF-α polyclonal antibody (Thermo Fisher Scientific) at a 1:500 dilution in 3% nonfat skim milk in TBS overnight at 4°C. Membranes were washed three times with TBST, followed by incubation with a 1:5,000 dilution of goat anti-rabbit IgG-alkaline phosphatase secondary antibody (Thermo Fisher Scientific) for 1 h at RT. Blots were washed three times with TBS and developed using SIGMAFAST BCIP/NBT (5-bromo-4-chloro-3-indolylphosphate–nitroblue tetrazolium) tablet dissolved in water (Sigma-Aldrich). The experiment was performed in biological and technical duplicate.

### Disk diffusion assay.

Sensitivity to oxidative stress and the corresponding catalase response were assessed using a hydrogen peroxide diffusion assay, as previously described (27, 57). Fungal cells were processed according to the infection conditions stated above and collected in 1 mL cold water. To lyse macrophages, the samples were briefly vortexed, followed by centrifugation at 3,500 rpm for 10 min. Cells were washed twice in 1× PBS, and cell density was counted using a hemocytometer and normalized to 2.5 × 10^5^ fungal cells. Cells were plated using cotton swabs on YPD semisolid medium plates supplemented with 34 μg/mL chloramphenicol for infection conditions containing both fungal and bacterial pathogens and antibiotic-free plates for sole-fungal-infection samples. Sterile paper discs (6.6 mm diameter) saturated with 10% H_2_O_2_ were added to the center of the plate. The plates were incubated at 37°C for 48 h and then photographed, and measurements were taken from three sides to the nearest millimeter to determine the radius of each zone of inhibition. In each case, a C. neoformans WT control grown in YPD medium at 37°C overnight and subcultured to mid-log phase was prepared as previously described. Experiments were completed in biological quadruplicate and technical duplicate.

### Melanin plate assay.

To examine the rate of C. neoformans ability to produce melanin pigmentation following conditioning under the multiple-infection conditions, cells were collected as described above, serially diluted 10-fold (10^4^ cells/5 μL) in 1× PBS, plated on minimal medium (29.4 mM KH_2_PO_4_, 10 mM MGSO_4_^−^·7H_2_O, 13 mM glycine, 3 μM thiamine, 0.27% dextrose) agar containing 1 mM l-DOPA (Sigma-Aldrich) supplemented with 34 μg/mL chloramphenicol, and incubated at 37°C for 6 days, with imaging every 24 h. For each infection condition, a C. neoformans WT control was additionally plated, grown overnight at 37°C in YPD, followed by overnight subculture in yeast nitrogen base (YNB) (Sigma-Aldrich), and then grown overnight in minimal medium for 16 h and prepared as described previously. To quantify melanin production *by* C. neoformans, we measured the percentage of pigment, as previously described (58). Briefly, each image was converted into an 8-bit grayscale, with a set area of 50 by 50 pixels to measure the gray values (range, 0 to 255). These values were converted into a percentage of pigment using the formula [(255 subtracted by the mean grayscale value for each spot)/255] × 100.

Next, values were normalized against the WT values for each plate. Experiments were completed in biological quadruplicate and technical triplicate.

### Data availability.

The RAW and affiliated files were deposited into the publicly available PRIDE partner database for the ProteomeXchange consortium with the data set identifier PXD033393.
